# THE IMPACT OF COVID-19 ON RESIDENTS AND FAMILY/FRIEND CAREGIVERS IN ASSISTED LIVING HOMES

**DOI:** 10.1093/geroni/igad104.1626

**Published:** 2023-12-21

**Authors:** Matthias Hoben, Colleen Maxwell, Anna Beeber

## Abstract

Healthcare reforms have neglected assisted living (AL) and nursing homes (NHs) for decades, setting them up for the excessive rates of death and suffering during COVID-19. Research before and during the pandemic has primarily focused on NHs, largely overlooking AL. AL is rapidly expanding, caring for people with similar vulnerabilities as those in NHs, yet is less regulated, offers fewer services, has lower staffing/skill mix levels and requires significant family/friend involvement in care. This symposium presents a program of research (COVCARES, COVID-19 and the Care of Assisted living Residents), aiming to understand how the pandemic has impacted AL resident, family/friend, and facility outcomes, and how resident outcomes compare between NHs and AL. Our research started over a decade ago with the first population-based cohort study comparing AL and NHs in Canada. Our current research includes repeated surveys (10/2020–04/2021 and 07/2021–09/2021) with family/friend caregivers and AL facilities, and population-based clinical and health administrative data (2017-2021) from AL and NH residents in Western Canada. Five presentations will report on the design/methods/goals of COVCARES (#1), the impact of the pandemic on family/friend involvement in AL resident care (#2), impacted of the pandemic on psychotropic drug prescriptions in AL (#3), and the association of AL home preparedness for and response to the pandemic on resident pain (#4) and loneliness (#5). Our discussant (Anna Beeber) will highlight similarities and differences between AL and NHs, similarities and differences in both settings between the US and Canada, and how policymakers can account for these differences.

